# Improved Adhesion, Growth and Maturation of Vascular Smooth Muscle Cells on Polyethylene Grafted with Bioactive Molecules and Carbon Particles

**DOI:** 10.3390/ijms10104352

**Published:** 2009-11-20

**Authors:** Martin Parizek, Nikola Kasalkova, Lucie Bacakova, Petr Slepicka, Vera Lisa, Martina Blazkova, Vaclav Svorcik

**Affiliations:** 1 Centre for Cardiovascular Research, Institute of Physiology, Academy of Sciences of the Czech Republic / Videnska 1083, CZ-14220 Prague 4, Czech Republic; E-Mail: parizek@biomed.cas.cz (M.P.); 2 Department of Solid State Engineering, Institute of Chemical Technology / Technicka 5, 16628 Prague 6, Czech Republic; E-Mails: Nikola.Kasalkova@vscht.cz (N.K.); Petr.Slepicka@vscht.cz (P.S.); Vaclav.Svorcik@vscht.cz (V.S.); 3 Department of Biochemistry and Microbiology, Institute of Chemical Technology / Technicka 5, 16628 Prague 6, Czech Republic; E-Mail: Martina.Blazkova@vscht.cz (M.B.)

**Keywords:** plasma irradiation, bioactivity, biocompatibility, tissue engineering and reconstruction

## Abstract

High-density polyethylene (PE) foils were modified by an Ar^+^ plasma discharge and subsequent grafting with biomolecules, namely glycine (Gly), polyethylene glycol (PEG), bovine serum albumin (BSA), colloidal carbon particles (C) or BSA and C (BSA + C). As revealed by atomic force microscopy (AFM), goniometry and Rutherford Backscattering Spectroscopy (RBS), the surface chemical structure and surface morphology of PE changed dramatically after plasma treatment. The contact angle decreased for the samples treated by plasma, mainly in relation to the formation of oxygen structures during plasma irradiation. A further decrease in the contact angle was obvious after glycine and PEG grafting. The increase in oxygen concentration after glycine and PEG grafting proved that the two molecules were chemically linked to the plasma-activated surface. Plasma treatment led to ablation of the PE surface layer, thus the surface morphology was changed and the surface roughness was increased. The materials were then seeded with vascular smooth muscle cells (VSMC) derived from rat aorta and incubated in a DMEM medium with fetal bovine serum. Generally, the cells adhered and grew better on modified rather than on unmodified PE samples. Immunofluorescence showed that focal adhesion plaques containing talin, vinculin and paxillin were most apparent in cells on PE grafted with PEG or BSA + C, and the fibres containing α-actin, β-actin or SM1 and SM2 myosins were thicker, more numerous and more brightly stained in the cells on all modified PE samples than on pristine PE. An enzyme-linked immunosorbent assay (ELISA) revealed increased concentrations of focal adhesion proteins talin and vinculin and also a cytoskeletal protein β-actin in cells on PE modified with BSA + C. A contractile protein α-actin was increased in cells on PE grafted with PEG or Gly. These results showed that PE activated with plasma and subsequently grafted with bioactive molecules and colloidal C particles, especially with PEG and BSA + C, promotes the adhesion, proliferation and phenotypic maturation of VSMC.

## 1. Introduction

Synthetic polymers, such as polyethylene, polystyrene, polyurethane, polytetrafluoroethylene and polyethyleneterephthalate, are commonly used in various industrial applications, as well as in biotechnology and medicine. These materials not only serve as growth supports for cell cultures *in vitro*, but can also be used for constructing replacements for various tissues or organs, *e.g.*, non-resorbable or semi-resorbable vascular prostheses, artificial heart valves, bone and joint replacements, and implants for plastic surgery (for reviews, see [1–3]).

Materials designed for the construction of body implants must be biocompatible, *i.e.*, matching the mechanical properties of the replaced tissue and not acting as cytotoxic, mutagenic or immunogenic. Biocompatible materials can also behave as bioinert, *i.e.*, not promoting cell adhesion and proliferation. For example, such types of materials have been applied in the construction of artificial eye lenses and in articular surfaces of joint prostheses, *i.e.*, implants requiring transparency or smoothness, and thus completely cell-free surfaces. Bioinert materials have also been used for fabricating polymeric vascular prostheses in order to prevent adhesion and activation of thrombocytes and immunocompetent cells on the inner surface of these grafts (for reviews, see [2,4]).

An alternative and more advanced approach, widely accepted in recent tissue engineering, is to create surfaces that support colonization with cells and good integration of a replacement with the surrounding tissues of the patient’s organism. This concept is used *e.g.*, for constructing bone prostheses that will persist in the patient’s organism for many years, and is being developed for the creation of bioartificial replacements of blood vessels, liver, pancreas and even nervous system tissue. In these replacements, the artificial materials have a similar function as the natural extracellular matrix, and they serve as templates for regeneration of the damaged tissue. For example, in vascular tissue engineering, such a material should enable reconstruction of the *tunica intima*, formed by a confluent layer of endothelial cells, and also reconstruction of the *tunica media* containing vascular smooth muscle cells. For such purposes, advanced artificial materials cannot merely be passively tolerated by cells, but should act as bioactive or biomimetic, *i.e.,* inducing the required cell responses in a controllable manner (for reviews, see [2–4]).

The cell-material interaction is strongly dependent on the physical and chemical properties of the material surface. The main properties decisive for colonization of a material with cells are polarity, wettability, electrical charge, roughness and topography. No less important is the presence of various chemical functional groups and biomolecules on the surface of these materials (for reviews, see [4,5]).

Unfortunately, many potential materials for the construction of tissue replacements have properties that are not so appropriate for integration with the surrounding tissues, and they need further modification in order to enhance their attractiveness for cell colonization and new tissue formation. The synthetic polymers mentioned above are a typical example. In their pristine state, these materials are characterized by relatively high hydrophobicity, *i.e.*, the water drop contact angle on their surface is higher than 90° (for reviews, see [4,5]).

There are various ways of modifying the surfaces of polymers to make them convenient for cell adhesion. For this purpose, the surfaces have been exposed to ultraviolet (UV) irradiation [6,7], to a beam of various ions (*e.g.*, oxygen, nitrogen, noble gases or halogens for biological applications [1–3,8]) or to a plasma discharge [9–12]. For more pronounced changes in the physicochemical properties of the modified surface, some of these processes can be realised in a gas atmosphere, *e.g.*, in acetylene or ammonia [6,7,13]. These modifications change the stability, roughness, morphology, mechanical properties and chemical composition of the polymer surface by creating chemical functional groups containing oxygen or nitrogen, *e.g.*, carbonyl, carboxyl or amine groups, on the surface of the material. These groups increase the surface wettability, support the adsorption of cell adhesion-mediating extracellular matrix proteins in an appropriate geometrical conformation and stimulate cell adhesion and growth [1–3,7].

An alternative and more exact approach for polymer surface modification can be to graft the polymer surfaces directly with various biomolecules, *e.g.*, with amino-acids, oligopeptides or protein molecules, which can influence the cell behavior in a more controllable manner. This grafting occurs spontaneously by exposing the material to biological environments, such as solutions of various biomolecules, blood, intercellular liquid or cell culture media, immediately after plasma activation. The biomolecules then bind to radicals created on the polymer surface by plasma activation [11,12].

Therefore, in this study, high-density polyethylene, a model material for potential biomedical use, was modified by an Ar^+^ plasma discharge and subsequent grafting of glycine (Gly), bovine serum albumin (BSA), polyethyleneglycol (PEG), colloidal carbon particles (C) or BSA + C. The aim of these modifications was to create surfaces attractive for cell colonization. On the modified polymer, we evaluated the adhesion, proliferation and phenotypic maturation of vascular smooth muscle cells in cultures derived from rat aorta.

## 2. Results and Discussion

### 2.1. Physicochemical Properties of Polymer Samples

**Figure 1.**
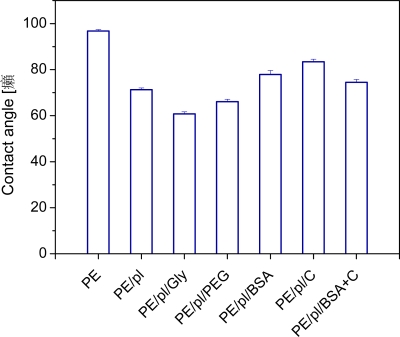
The contact angle of pristine PE (PE), plasma-treated PE (PE/pl), PE plasma-treated and then grafted with glycine (PE/pl/Gly), PEG (PE/pl/PEG), BSA (PE/pl/BSA), C (PE/pl/C) or BSA + C (PE/pl/BSA + C).

It is well known that chemical structure and surface morphology have a significant effect on surface wettability [12,14,15], which in turn may affect adhesion and proliferation of living cells [12,14]. The contact angle, as a measure of surface wettability, is shown in Figure 1 for pristine PE, plasma treated PE and plasma-treated and biomolecule-grafted samples. The contact angle decreases for samples treated by plasma, which is mainly related to the formation of oxygen structures during plasma-irradiation [16]. Obviously, the oxygen from the ambient residual atmosphere interacts with the plasma activated PE surface, and creates various oxidized structures. Creation of carbonyl, carboxyl and ester groups on plasma-treated PE was proven earlier in similar experiments [17,18]. A further decrease in the contact angle is obvious after glycine and PEG grafting, where the hydrophilicity of the sample increases in comparison to PE treated only with plasma. This finding supports the idea that the polar molecules (Gly, PEG) are linked to the activated PE chain [14]. By contrast, after BSA, C and BSA + C grafting, the values of the contact angle were higher than after plasma treatment, but lower than in the case of pristine PE. In our previous studies, covalent grafting of the mentioned molecules to the polymer surface was proved by the decrease in the concentration of radicals and double bonds on the modified polymer surface [19,20].

**Figure 2.**
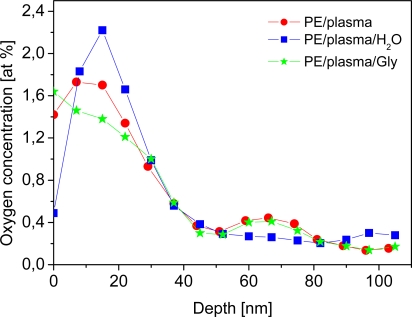
Depth concentration profile of oxygen, determined from RBS measurement, on the sample exposed to plasma discharge (PE/plasma), plasma-treated sample immersed in water (PE/plasma/H_2_O) and plasma-treated sample grafted with glycine (PE/plasma/Gly).

The presence of oxygen in the surface layer of the plasma-treated PE was examined using the RBS method. The oxygen depth concentration profile on the polymer surface is shown in Figure 2 for plasma-treated PE, plasma-treated PE immersed in water, and plasma-treated PE grafted with glycine. Water-treating experiments were performed because all the biomolecules and C were grafted from a water solution, and then the samples were exposed to a water-based cell culture environment. Thus, it was necessary to know what influence the water has on the plasma-treated PE surface. It is apparent that oxygen is present not only on the very surface of the sample, but also in the near surface layer about 50 nm in thickness. The oxygen concentration reached its maximum at a depth of about 20 nm, and then decreased to the bulk. The presence of oxygen beneath the PE surface may indicate an inward diffusion of oxygen, which could be facilitated by the structure of the PE surface altered by the preceding plasma treatment. The results given in Figure 2 indicate that water-treating of plasma-treated PE leads to a dramatic decrease in oxygen concentration in the PE surface layer, caused by dissolution in water of the polymer macromolecules, degraded by plasma irradiation [21]. On the other hand, the increase in oxygen concentration after glycine grafting proves that this amino-acid was chemically linked to the plasma-activated surface [12]. The oxygen concentration in plasma-treated PE, the plasma-treated and water-immersed sample, and the plasma-treated and PEG-grafted sample is shown in Table 1. Similarly as in Figure 2, it is evident that the oxygen concentration decreases after water-treating. After PEG grafting, however, the oxygen concentration increases compared to the water-etched sample, which proves that there is chemical linking of the oxygen compound on the activated polymer chain. In pristine PE, no oxygen content was detected by RBS measurement. Also pristine PE exposed to water, PEG or Gly was oxygen-free. There are no radicals and double bonds on a pristine PE surface, and therefore it is not possible to graft PEG and Gly to this material.

**Figure 3.**
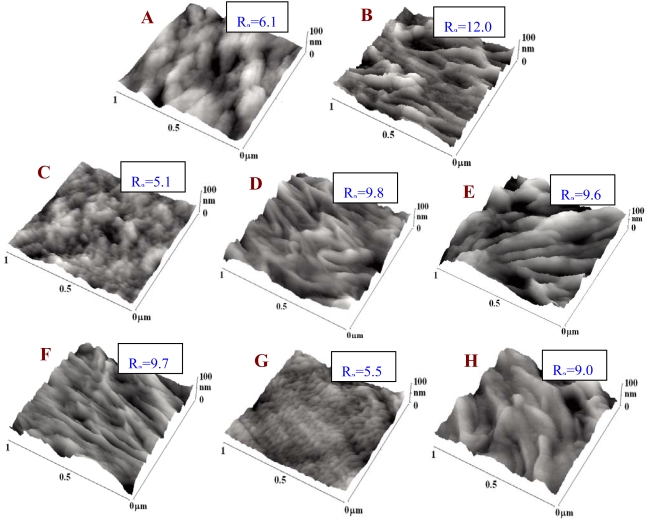
AFM images of pristine PE (A), plasma-treated PE (B), PE treated with plasma and immersed in water (C), PE treated with plasma and grafted with glycine (D), PEG (E), BSA (F), colloidal carbon particles (G) or BSA + C (H). R_a_ is a parameter of measured surface roughness in nm.

The surface morphology of pristine PE and PE exposed to a plasma discharge and then etched or grafted was examined using the AFM method (Figure 3). Plasma treatment led to ablation of the PE surface layer [17,18]. As a result, the surface morphology changed dramatically and the nanoscale surface roughness increased. Also a lamellar structure appeared on the PE surface, which could be due to the different ablation rates of the amorphous and crystalline phases [21]. Further changes in the surface morphology and a decrease in the surface roughness occurred after exposure of the samples to a water environment. These changes were probably due to preferential etching of short molecular fragments formed by the plasma treatment [18]. Figure 3 also demonstrates changes in the surface morphology and a further increase in surface roughness after grafting the plasma-activated PE with glycine, PEG, BSA and BSA + C. Interestingly, when we compare the water-treated samples and the samples grafted by molecules of very different molecule weights (*i.e.*, glycine *vs*. PEG), the increase in material surface roughness does not depend on the molecular weight of the grafted molecule.

### 2.2. Initial Adhesion of VSMC on Polymer Samples

**Figure 4.**
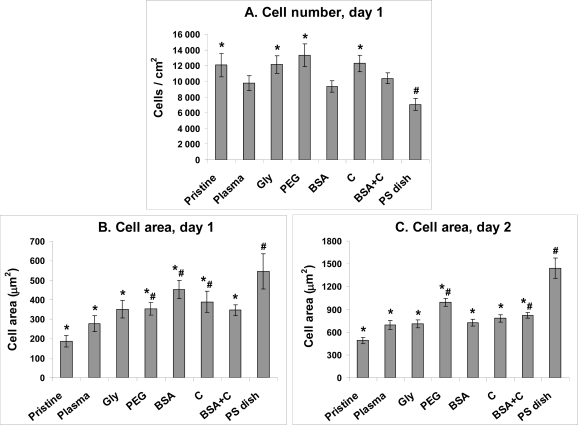
The number of initially adhered cells (A) and the size of the cell spreading area (B, C) of rat aortic smooth muscle cells in cultures on non-modified PE (Pristine), PE irradiated with plasma (Plasma), PE irradiated with plasma and grafted with glycine (Gly), polyethyleneglycol (PEG) bovine serum albumin (BSA), colloidal carbon particles (C) or bovine serum albumin and C (BSA + C). As a reference material, a tissue culture polystyrene dish (PS dish) was used. Means ± S.E.M. from 3 samples, each measured for 50 times (A) or from 72 to 142 cells for each experimental group (C, D). ANOVA, Student-Newman-Keuls method. Statistical significance: ^*, **#**:^ p ≤ 0.05 compared to the value on pristine PE and polystyrene dish, respectively.

On day 1 after seeding, the numbers of initially adhered cells on all PE samples, including those unmodified, ranged between 9,340 ± 740 to 13,310 ± 1,430 cells/cm^2^, while on the control PS, only 7,060 ± 770 cells/cm^2^ were found. The highest cell numbers, significantly higher than those on PS, were found on PE grafted with PEG (13,310 ± 1,430 cells/cm^2^), C (12,280 ± 1,070 cells/cm^2^) and Gly (12,130 ± 1,130 cells/cm^2^, Figure 4A). This was a favourable result, because tissue culture polystyrene (*i.e.*, PS usually modified by a glow discharge, *i.e.,* a similar technique as the plasma-irradiation used in our study) is considered as an ideal material for cell adhesion and proliferation. However, it is generally known that irradiation of polymers with a plasma discharge improves cell adhesion and growth. This beneficial effect has been attributed to the creation of oxygen-containing functional groups on the polymer surface. Fourier Transform Infrared Spectroscopy (FTIR) has indicated the presence of peroxide, ester, carbonyl, carboxyl, hydroxyl and amide groups, as well as excessive double bonds in polyethylene modified with a plasma discharge [21]. Also in this study, RBS proved an increase in oxygen concentration in PE after plasma treatment and plasma treatment followed by grafting Gly or PEG molecules, *i.e.*, donors of additional oxygen-containing groups (Table 1, Figure 2). These groups are known to increase surface wettability and improve the adsorption of cell adhesion-mediating extracellular matrix molecules (*e.g.*, vitronectin, fibronectin) from the serum of the culture medium. These molecules are adsorbed in an appropriate and flexible spatial conformation, enabling good accessibility of specific sites on these molecules (*e.g.*, RGD-containing oligopeptides) by cell adhesion receptors, such as integrins (for a review, see [2,4,5]). A similar effect has been described for nanostructured surfaces [22]. Therefore, from this point of view, the nanostructured surfaces act synergetically with oxygen-containing and wettable surfaces. As revealed by AFM, the nanoscale surface roughness of PE in our study became more apparent after plasma irradiation, and was also retained in a water environment and after grafting the polymer with biomolecules and C (Figure 3).

### 2.3. Spreading of VSMC on Polymer Samples

The cells on the modified PE samples were better spread, *i.e.*, adhering by a larger cell-material projected area, than on pristine PE (Figure 4B, C). On the 1^st^ day after seeding, the largest spreading areas were found in cells adhering to the polystyrene dishes (546 ± 90 μm^2^), and PE grafted with BSA (453 ± 47 μm^2^), C (390 ± 53 μm^2^) or PEG (354 ± 32 μm^2^), whereas on non-modified PE the value was only 187 ± 29 μm^2^. On day 2 after seeding, the largest cell spreading area was again achieved on the polystyrene dishes (1440 ± 137 μm^2^) and on PE grafted with PEG (993 ± 52 μm^2^), compared to only 492 ± 42 μm^2^ in VSMC on pristine PE. Also in our earlier studies, PE in its pristine non-modified form proved to be a substrate not very suitable for cell adhesion and spreading [8,11,14]. This behaviour of pristine PE can be attributed to its relatively high hydrophobicity, characterized by a water drop contact angle of almost 100° (Figure 1) compared with 102.5 ± 2.3° found in our earlier study [21], and also to the lack of oxygen-containing groups.

### 2.4. Proliferation of VSMC on Polymer Samples

**Figure 5.**
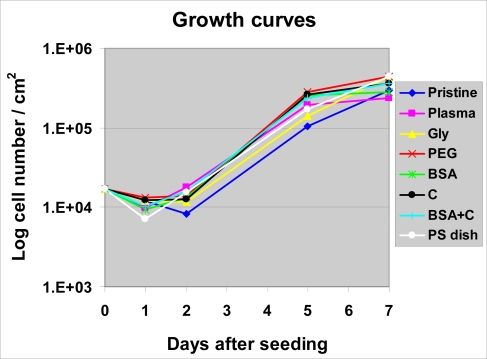
Growth dynamics of rat aortic smooth muscle cells in cultures on non-modified PE (Pristine), PE irradiated with plasma (Plasma), PE irradiated with plasma and grafted with glycine (Gly), polyethyleneglycol (PEG) bovine serum albumin (BSA), colloidal carbon particles (C) or bovine serum albumin and C (BSA + C). As a reference material, a tissue culture polystyrene dish (PS dish) was used. Means from three samples for each experimental group and time interval (each sample measured for 50 times).

**Figure 6.**
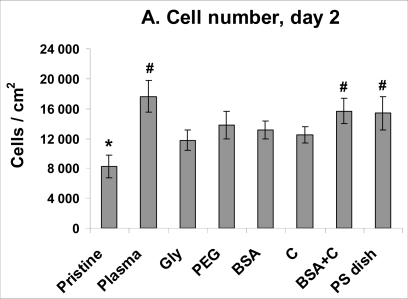

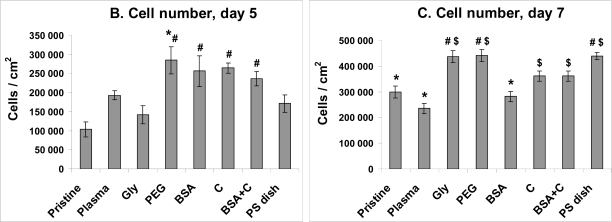
Proliferation activity of rat aortic smooth muscle cells measured by cell numbers achieved on day 2 (A), 5 (B) and 7 (C) after seeding on non-modified PE (Pristine), PE irradiated with plasma (Plasma), PE irradiated with plasma and grafted with glycine (Gly), polyethyleneglycol (PEG) bovine serum albumin (BSA), colloidal carbon particles (C) or bovine serum albumin and C (BSA + C). As a reference material, a tissue culture polystyrene dish (PS dish) was used. Means ± S.E.M. from three samples for each experimental group, each measured for 50 times. ANOVA, Student-Newman-Keuls method. Statistical significance: ^*,#, $^: p ≤ 0.05 compared to the value on pristine PE, polystyrene dish and PE irradiated with plasma, respectively.

Despite a lower number of initially adhered cells on PS, on day 2 after seeding, the cells on PS were in the exponential phase of growth, while the cells on most PE samples still remained in the lag phase (except plasma-irradiated PE and PE grafted with BSA + C, Figure 5). As a result, the cell number on PS (15,370 ± 2,240 cells/cm^2^) became significantly higher than that on non-modified PE (8,240 ± 1,500 cells/cm^2^) and similar to the values found on all modified PE samples. Among these, the highest cell population densities were obtained on PE irradiated with plasma (17,650 ± 2,080 cells/cm^2^) and subsequently grafted with BSA and C (15,660 ± 1,690 cells/cm^2^), and these values were significantly higher than that on pristine PE (Figure 6A).

On day 5 after seeding, the highest cell numbers were obtained on samples modified with PEG (285,000 ± 35,000 cells/cm^2^), C (265,000 ± 14,000 cells/cm^2^), BSA (256,000 ± 40,000 cells/cm^2^) and BSA + C (236,000 ± 19,000 cells/cm^2^). On PE grafted with PEG, the cell number was even significantly higher than that on PS (171,000 ± 23,000 cells/cm^2^). The lowest number of cells persisted on pure PE (104,000 ± 19,000 cells/cm^2^, Figure 6B).

On day 7, the cell numbers on PE grafted with PEG (442,000 ± 24,000 cells/cm^2^) and Gly (436,000 ± 24,000 cells/cm^2^) became significantly higher, not only in comparison with pristine PE (299,000 ± 23,000 cells/cm^2^), but also in comparison with PE modified only with plasma irradiation (235,000 ± 20,000 cells/cm^2^). Also on PE grafted with C and BSA + C, the cell numbers were significantly higher than on plasma-irradiated PE. On the latter sample, the cell number was significantly lower than that found on polystyrene culture dishes (440,000 ± 135,000 cells/cm^2^; Figure 6C). As documented by the growth curves, from day 5 to 7, the cells on the plasma-modified samples entered the stationary phase, *i.e.,* did not significantly change their number (Figsures 5, 6B, C). A usual explanation for such cell behaviour is that confluence of the cells is attained and their growth ceases due to contact inhibition. However, as mentioned above, the cells in other materials were able to continue their growth, though more slowly. VSMC are able to form multilayered regions *in vitro*, referred to as “hills” [22], thus they become contact-inhibited at relatively high population densities. Another explanation for the cessation of VSMC growth on the plasma-modified PE could be that the plasma irradiation did not sufficiently increase the wettability of PE, which still remained lower than that of the cell culture polystyrene. The contact angle of traditional tissue culture polystyrene has been reported to range between 55.8° and 63.5° [24], while the plasma-irradiated PE in our study reached a higher value of about 70° (Figure 1). The concentration of oxygen, which is also supportive for cell adhesion and proliferation, was 17.2 % in tissue culture PS as measured by the Electron Spectroscopy for Chemical Analysis (ESCA) [24], whereas in plasma-treated PE, it was only about 0.5 at.% immediately on the polymer surface (which enters into contact with adsorbed proteins and attached cells), and about 1.7 at a depth of 10–20 nm (Figure 2). As mentioned above, the exposure of plasma-treated PE to a water environment used for grafting biomolecules (and also for cell cultivation), caused a further decrease in the oxygen content in the surface layer of the polymer. Last but not least, plasma modification is known to create radicals on the polymer surface [21]; for reviews, see [5,12], which can damage the cells and thus they can hamper the process of cell adhesion and proliferation.

**Figure 7.**
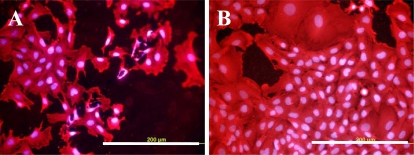

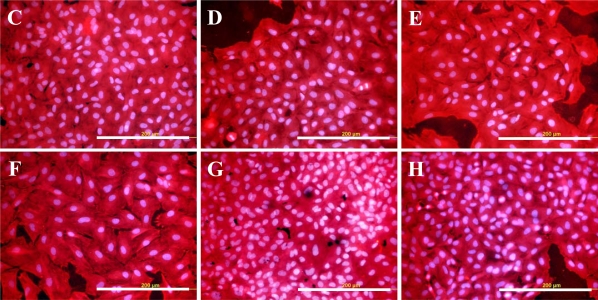
Morphology of rat aortic smooth muscle cells on day 5 after seeding on pristine PE (A), tissue culture polystyrene dish (B), PE irradiated with Ar^+^ plasma (C), PE irradiated with plasma and grafted with glycine (D), polyethyleneglycol (E) bovine serum albumin (F), colloidal carbon particles (G) or bovine serum albumin and C (H). Cell membrane and cytoplasm stained with Texas Red C2-maleimide (red fluorescence), cell nuclei with Hoechst #33342 (blue fluorescence). Olympus IX 51 microscope, DP 70 digital camera, obj. 20×, bar = 200 μm.

On the other hand, the radicals present on the surface of plasma-irradiated polymers can be efficiently used for grafting biomolecules. The results of this study proved that grafting PE with biomolecules, such as glycine, PEG, BSA and/or carbon particles, can create surfaces which are suitable for the adhesion and growth of VSMC. On all grafted samples, the cells were able to create confluent layers at the end of the experiment, *i.e.*, on day 7 after seeding (Figure 7). All modified PE samples seemed not to have any considerable cytotoxic effects, as suggested by the numbers of viable cells determined by the ViCell Analyser, which ranged between 73 and 97%. The number of viable cells increased from day 1 to day 7 of cultivation, which suggests that the cells were damaged by trypsinization and the counting procedure rather than by exposure to the modified PE samples.

The increase in the cell number and the size of the cell spreading area from day 1 to 7 was usually well-apparent on PE samples grafted with PEG or BSA. In other studies, however, both PEG and BSA have often been reported to be non-adhesive for cells and have even been used for constructing inert cell non-adhesive surfaces (for a review, see [25]). The anti-adhesive action of PEG is based on its very high hydrophilia and the mobility of its chain, which hamper stable adsorption of the proteins that mediate cell adhesion. However, at the same time, the anti-adhesive action of PEG is dependent on its concentration on the polymer surface and on the length of its chain. In our earlier studies performed on copolymers of poly(dl-lactide) (PDLLA) and PEG, the negative effect of PEG on the adhesion and proliferation of VSMC was most pronounced at intermediate concentrations of the PEG phase on the surface of the polymeric film (33–45 wt.%) and also at an intermediate length (m.w. 11,000) of the PEG chains. Both lower and higher concentrations (*i.e.*, 5 to 18 wt.% and 70 wt.%, respectively) and lengths (m.w. 5,000 and 23,800, respectively) permitted the attachment, spreading and formation of vinculin-containing focal adhesion plaques in VSMCs to an extent comparable to that observed on pure PDLLA and standard cell culture substrates, such as polystyrene dishes or microscopic glass coverslips [25,26]. Also the PEG chains used in the present study were rather long, having a molecular weight of 20,000.

As for BSA, this protein cannot be directly bound by cells. However, it has been reported to promote the adsorption of vitronectin and fibronectin in advantageous spatial conformations, supporting the accessibility of these molecules by cell adhesion receptors [27,28]. These molecules can be adsorbed over the albumin from the serum of the culture medium, and also synthesized and deposited by VSMCs themselves. When adsorbed on albumin, these molecules promote cell adhesion even if their concentrations are too low to support cell attachment alone [29].

The cell adhesion and proliferation on PE grafted with BSA was often improved after additional grafting of C. In our earlier studies, and also in studies by other authors, enrichment of cell adhesion substrate with carbon, *e.g.*, after ion irradiation [2,30], coating with amorphous carbon [31] or by deposition of carbon nanoparticles [32] (for a review, see [5]) often resulted in enhanced adhesion and growth of cells in cultures on these materials.

Grafting with glycine also improved the cell colonization of PE in the present study. This amino acid is a component of RGD-containing oligopeptides, which act as ligands for cell adhesion receptors. Although Gly alone cannot directly bind these receptors, it provides the grafted polymer surface with additional oxygen-containing groups and positively charged amine groups, which improve the adsorption of cell adhesion-mediating molecules from the serum of the culture medium [6,13,33].

### 2.5. Distribution and Concentration of Molecular Markers of Adhesion and Maturation in VSMCs

**Figure 8.**
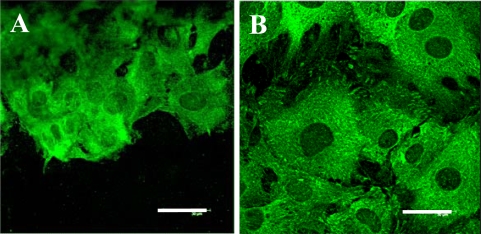

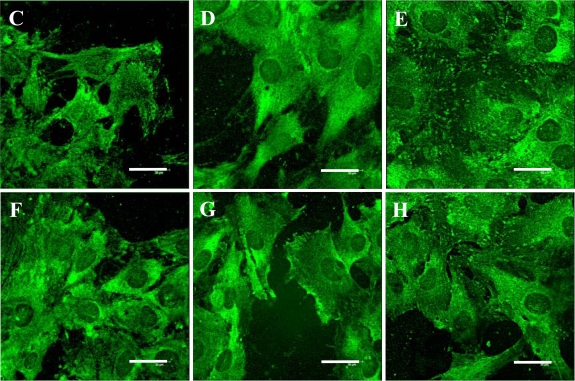
Immunofluorescence staining of talin, an integrin-associated protein of focal adhesion plaques, in rat aortic smooth muscle cells on day 5 after seeding on pristine PE (A), a reference material represented by a microscopic glass coverslip (B), PE irradiated with plasma (C), PE irradiated with plasma and grafted with glycine (D), polyethyleneglycol (E) bovine serum albumin (F), colloidal carbon particles (G) or bovine serum albumin and C (H). Leica confocal laser scanning microscope (TCS SP2, Germany), obj. 100x, bar = 30 μm.

In accordance with the data obtained on the cell number and the cell spreading area, immunofluorescence staining revealed that focal adhesion plaques containing talin, vinculin and paxillin were generally better developed in cells on modified PE samples, particularly those grafted with PEG and BSA + C, than in cells on pristine PE samples (Figure 8). ELISA showed that the cells on PE modified with PEG or BSA + C contained higher concentrations of talin, a protein associated with integrin adhesion receptors (about twice compared to pristine PE and about 1.5 times compared to PE modified only by plasma irradiation, Table 2). The concentration of vinculin, another protein of focal adhesion plaques associated with cell adhesion receptors, in cells on PE with BSA + C was almost 1.9 and 1.7 times higher than in cells on pristine PE and PS dishes, respectively (Table 2). The concentration of focal adhesion protein paxillin was similar in cells on all studied samples. However, the concentration of β-actin, a protein of the cytoplasmic cytoskeleton associated with focal adhesions and thus an important marker of cell adhesion and spreading, was increased by 1.7 to 2 times in cells grown on PE modified with PEG, BSA, C and BSA + C compared to cells on PS dishes (Table 2).

**Figure 9.**
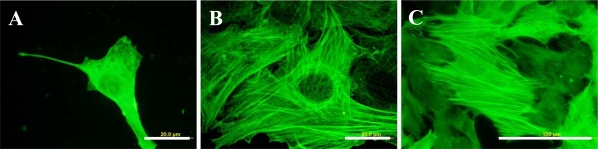

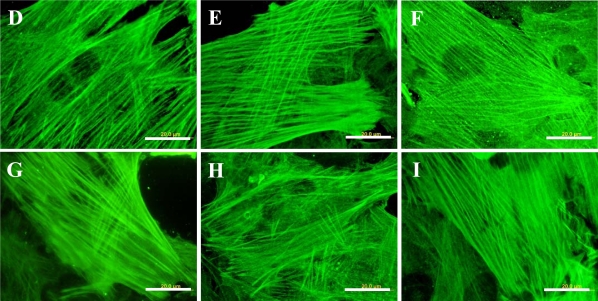
Immunofluorescence staining of contractile protein alpha-actin in rat aortic smooth muscle cells on day 5 after seeding on pristine PE (A), reference materials represented by a microscopic glass coverslip (B), a standard cell culture polystyrene dish (C), PE irradiated with plasma (D), PE irradiated with plasma and grafted with glycine (E), polyethyleneglycol (F), bovine serum albumin (G), colloidal carbon particles (H) or bovine serum albumin and C (I). Olympus IX 51 microscope, DP 70 digital camera, obj. 100× (A, B, D-I) or 40× (C). Bar = 20 μm (A, B, D-I) or 100 μm (C).

Immunofluorescence also revealed that fibres containing cytoskeletal protein β-actin, and particularly α-actin or SM1 and SM2 myosins, *i.e.*, markers of phenotypic maturation of VSMCs toward a more differentiated contractile phenotype [22,34], were thicker, more numerous and more brightly stained in cells on all modified PE samples than on pristine PE (Figure 9). The concentration of α-actin, measured by ELISA per mg of protein, was significantly increased in cells on the polymer grafted with PEG (by about 1.4 times compared to polystyrene and plasma-modified PE) or with Gly (1.3 times compared to PS, Table 2). The highest average concentration of SM1 and SM2 myosins was found in cells growing on PE with PEG (128% of the value found on pristine PE), but this difference did not reach statistical significance (Table 2). The concentration of α-actinin, a protein associated with α-actin filaments, was the highest in cells cultured on plasma-modified PE. In these samples, it was even significantly higher than in cells on other modified samples, namely with Gly (1.6 times), PEG (1.5 times) and BSA (2.5 times, Table 2).

It should be pointed out that the conventional ELISA applied in this study measured the total numbers of the investigated molecules in the cells, *i.e.*, not only those bound in focal adhesion plaques or cytoskeletal and contractile fibres, but also those present in cytoplasm and organelles, such as the Golgi complex. Selective (and thus more precise) quantification of the investigated molecules in the specific adhesive, cytoskeletal and contractile structures could be achieved by more specialized approaches, *e.g.*, by using antibodies against phosphorylated antigens or by extraction of molecules non-bound in the membrane or cytoplasmatic cytoskeleton by detergents, such as Triton [35,36]. Nevertheless, the results of this study show that activation of PE with plasma and subsequent grafting with bioactive molecules and atoms stimulates the molecular mechanism, leading to enhanced adhesion and maturation of vascular smooth muscle cells in cultures on these materials.

## 3. Experimental Section

### 3.1. Preparation of the Polymer Samples

The experiments were carried out on high-density polyethylene foils (HDPE, PE) of the Microten M*S type (thickness 0.04 mm, density 0.951 g.cm^−3^, melt flow index 0.14 g/10 minutes), purchased from Granitol a.s., Moravsky Beroun, Czech Republic. The foils were cut into circular samples (2 cm in diameter) using a metallic perforator. The samples were then modified by an Ar^+^ plasma discharge (gas purity 99.997%) using a Balzers SCD 050 device. The time of exposure was 50 seconds, and the discharge power was 1.7 W. Immediately after plasma modification, the samples were immersed in water solutions of glycine (Gly; Merck, Darmstadt, Germany, product No. 104201), bovine serum albumin (BSA; Sigma-Aldrich, Germany, product No. A9418) or polyethyleneglycol (PEG; Merck, Darmstadt, Germany, product No. 817018, m.w. 20,000). Some plasma-treated samples and samples grafted with BSA were exposed to a suspension of colloidal carbon particles (C; Spezial Schwartz 4, Degussa AG, Germany) [37]. Each substance was used in a concentration of 2 wt.%, and the time of immersion was 12 hours at room temperature (RT).

### 3.2. Evaluation of the Physical and Chemical Properties of the Polymer Samples

Surface wettability was measured by goniometry, *i.e.*, the static (sessile) water drop contact angle method. It was shown that the contact angle of PE (exposed to plasma discharge) depends on the time elapsed from the moment of exposure [21]. Therefore, in this study, the contact angle on the plasma-modified and grafted samples was measured 20 days after the modification. The measurements of the advancing water contact angles (error ± 5%) were performed in 10 different positions at room temperature on the Surface Energy Evaluation System (Advex Instruments, Czech Republic) [21,31].

The concentration and depth concentration profile of oxygen in the modified PE surface layer was proven by Rutherford Backscattering Spectroscopy (RBS). The RBS analysis was performed in a vacuum target chamber with 2.72 MeV He^+^ ions. The elemental depth profiles in the inspected polymeric samples were determined with typical depth resolution less than 10 nm, and with an accessible depth of a few μm. The RBS spectra were evaluated by the GISA3.99 code [12]. The typical RBS detection limit is 0.1 at.% for oxygen.

The surface morphology and roughness of the pristine and modified samples were examined by the AFM technique, using a VEECO CP II device working in tapping mode. We used an RTESPA-CP Si probe, with spring constant 20–80 N/m. By repeated measurements of the same region (1 × 1 μm), it was proven that the surface morphology did not change after three consecutive scans. The mean roughness value (R_a_) represents the arithmetic average of the deviations from the centre plane of the sample.

### 3.3. Cell Source and Culture Conditions

For an evaluation of cell number, morphology and immunofluorescence staining, each modified round sample (2 cm in diameter) was cut into four smaller samples. For semiquantification of cell adhesion and differentiation-indicating molecules by an enzyme-linked immunofluorescence assay (ELISA), the samples were used in whole. Both smaller and bigger samples were sterilized with 70% ethanol for one hour and inserted into 24-well and 6-well plates (TPP, Switzerland; well diameter 1.5 cm and 3.5 cm, respectively), and air-dried for 12 hours in a sterile environment to prevent possible negative effects of alcohol on the cells. After drying, the samples were fixed to the bottom of the culture wells by plastic rings (inner area 0.38 cm^2^ and 4.91 cm^2^ for smaller and bigger samples, respectively) in order to prevent the samples floating in the cell culture media. After that, the samples were seeded with smooth muscle cells derived from rat aorta by an explantation method (passage 3, 17,000 cm^2^; [2,3]). The cells were cultivated in 1.5 mL Dulbecco’s Modified Eagle Minimum Essential Medium (Sigma, U.S.A.) supplemented with 10% foetal bovine serum (Sebak GmbH, Aidenbach, Germany) for 1, 2, 5 or 7 days (temperature of 37 °C, humidified atmosphere of 5% of CO_2_ in the air). For each experimental group and time interval, four smaller samples were used. ELISA was performed on day 7 only, and 6 samples were used for each experimental group.

### 3.4. Evaluation of the Cell Number and Morphology

On days 1, 2, 5 and 7 after seeding, the cells on the smaller polymer samples were rinsed in phosphate-buffered saline (PBS). The cells on one sample for each experimental group and time interval were fixed by 70% cold ethanol (−20 °C) and stained with a combination of fluorescent membrane dye Texas Red C_2_-maleimide (Molecular Probes, Invitrogen, Cat. No.T6008; concentration 20 ng/mL in PBS) and a nuclear dye Hoechst # 33342 (Sigma, U.S.A.; 5 μg/mL in PBS). The number, morphology and distribution of cells on the sample surface were then evaluated on pictures taken under an Olympus IX 51 microscope using an Olympus DP 70 digital camera. The pictures taken on days 1 and 2 after seeding were also used for measuring the size of the cell spreading area, using Atlas software (Tescan Ltd., Czech Republic).

The three remaining samples were the main material for evaluation of the cell number. The cells were rinsed by PBS, released with trypsin-EDTA solution (Sigma, Cat. No. T4174; incubation five minutes at 37 °C) and counted in a Cell Viability Analyzer (VI-cell XR, Beckman Coulter). During cell counting, this instrument performs an automatic analysis of the number of viable and dead cells, based on a trypan blue exclusion test.

### 3.5. Immunofluorescence Staining

On day 7 after seeding, the cells were rinsed twice in PSB, fixed with cold 70% ethanol (−20 °C, 15 min) and pre-treated with 1% bovine serum albumin in PBS containing 0.05% Triton X-100 (Sigma) for 20 minutes at room temperature in order to block non-specific binding sites and permeabilize the cell membrane. Next, the cells were incubated with primary antibodies against several molecular markers of adhesion and phenotypic maturation of VSMC, namely integrin-associated focal adhesion proteins talin, vinculin and paxillin, actin-binding protein alpha-actinin, cytoskeletal protein beta-actin and contractile proteins alpha-actin and SM1 and SM2 myosins (Table 3).

The antibodies were diluted in PBS to concentrations of 1:200 and applied overnight at 4 °C. After rinsing with PBS, the secondary antibodies (dilution 1:400) were added for 1 hour at room temperature. These antibodies were goat anti-mouse or goat anti-rabbit F(ab')2 fragments of IgG (H + L), both conjugated with Alexa Fluor® 488 and purchased from Molecular Probes, Invitrogen (Cat. No. A11017 and A11070, respectively). The cells were then rinsed twice in PBS, mounted under microscopic glass coverslips in a Gel/Mount permanent fluorescence-preserving aqueous mounting medium (Biomeda Corporation, Foster City, CA, U.S.A.), and evaluated under a Leica confocal microscope (TCS SP2, Germany; obj. HCX PL APO CS 100.0x1.40 OIL) and an Olympus IX 51 microscope (obj. 100x), using an Olympus DP 70 digital camera [2].

### 3.6. Enzyme-Linked Immunosorbent Assay (ELISA)

The concentration of the adhesion and differentiation molecules was measured semiquantitatively using an enzyme-linked immunosorbent assay (ELISA) in homogenized cells per mg of protein. On day 7 after seeding, the cells were rinsed with PBS, released with a trypsin-EDTA solution (Sigma, Cat. No. T4174, incubation for 5 minutes at 37 °C) and counted in a Cell Viability Analyzer (Vi-Cell XR, Beckman Coulter). Trypsinized cells were resuspended in PBS, centrifuged, resuspended in distilled and deionized water (10^6^ cells in 200 μL), and kept in a freezer at −70 °C overnight. The cells were then homogenized by ultrasonication for 10 seconds in a Bandelin Sonoplus HD 3080 sonicator (BANDELIN electronic GmbH & Co.), and the total protein content was measured using a modified method originally developed by Lowry [38]; modified by [2,39]. Aliquots of the cell homogenates corresponding to 1–50 μg of protein in 50 μL of water were adsorbed on 96-well microtiter plates (Maxisorp, Nunc) at 4 °C overnight. After washing twice with PBS (100 μL/well), the non-specific binding sites were blocked by 0.02% gelatin in PBS (100 μL/well, 60 minutes) and then treated by 1% Tween (Sigma, Cat. No. P1379; 100 μL/well, 20 minutes). The primary antibodies, the same as for immunofluorescence (Table 3), were diluted in PBS (1:200 to 1:500) and applied for 60 minutes at room temperature (50 μL/well). Secondary antibodies, *i.e.,* goat anti-mouse IgG Fab specific (dilution 1:1000), or goat anti-rabbit IgG whole molecule (dilution 1:1000), both conjugated with peroxidase and purchased from Sigma (Cat. No. A3682 and A0545, respectively), were applied for 45 minutes at room temperature (50 μL/well). This step was followed by a double washing in PBS and orthophenylendiamine reaction (Sigma, Cat. No. P1526, concentration 2.76 mM) using 0.05% H_2_O_2_ in 0.1 M phosphate buffer (pH 6.0, dark place, 100 μL/well). The reaction was stopped after 10–30 minutes by 2M H_2_SO_4_ (50 μL/well), and the absorbance was measured at 490 and 690 nm by a Versa Max Microplate Reader (Molecular Devices Corporation, Sunnyvale, California, USA). The absorbance of the cell samples taken from the modified PE foils was given as a percentage of the value obtained in cells on the pure PE [2,39].

### 3.7. Statistics

The quantitative results were presented as mean ±S.E.M. (Standard Error of Mean). Statistical analyses were performed using SigmaStat (Jandel Corp., USA). Multiple comparison procedures were made by the One Way Analysis of Variance (ANOVA), Student-Newman-Keuls method. *P* values equal to or less than 0.05 were considered significant.

## 4. Conclusions

Modifications of PE samples with a plasma discharge and subsequent grafting with biomolecules enhanced the colonization of PE with numerous and well-spread vascular smooth muscle cells with numerous talin- and vinculin-containing focal adhesion plaques. As suggested by the more numerous and thicker filaments containing a contractile protein alpha-actin, and also by the higher concentration of this protein per mg of protein, the cells on the modified polymers, particularly those grafted with PEG or Gly, also showed a higher level of phenotypic maturation. The beneficial effect of the plasma discharge could be attributed to the formation of oxygen-containing structures in the polyethylene surface layer, increased material wettability and changes in the surface morphology. Oxygen was present not only on the very surface of the sample, but also in the underlying surface layer about 50 nm in thickness. However, water-treating of plasma-irradiated PE led to a decrease in oxygen concentration in the surface layer, due to dissolution of plasma-degraded macromolecules in water. On the other hand, ablation of the surface layer by plasma-irradiation resulted in changes in the polymer surface morphology, and in an increase in the surface nanoscale roughness, which is considered to promote cell adhesion and growth. As indicated by grafting the polymer with glycine and PEG, the increase in surface roughness did not depend on the molecular weight of the grafted molecules. Grafting biomolecules (Gly, PEG, BSA) and colloidal C particles further increased the attractiveness of PE for VSMC colonization. As demonstrated on PE grafted with Gly and PEG, these biomolecules further increased the oxygen concentration in the material surface and the surface wettability. The supportive effect of biomolecules on cell colonization was most apparent on the polymer modified by PEG and BSA + C.

## Figures and Tables

**Table 1.** Surface oxygen concentration determined from RBS measurement in plasma-treated PE (PE/plasma), plasma-treated and water-immersed PE (PE/plasma/H_2_O), and plasma-treated PE then grafted with PEG (PE/plasma/PEG).

**Sample**	**O concentration (10^16^ at cm^−2^)**

PE/plasma	2.63
PE/plasma/H_2_O	1.23
PE/plasma/PEG	2.19

**Table 2.** Concentration of focal adhesion, cytoskeletal and contractile proteins in rat aortic smooth muscle cells in 7-day-old cultures on pristine PE (Pristine), PE activated with plasma (Plasma), PE grafted with glycine (Gly), polyethylene glycol (PEG), bovine serum albumin (BSA), colloidal carbon particles (C) or BSA with C (BSA + C), and tissue culture polystyrene dish (PS dish).

**Protein/PE modification**	**Talin**	**Vinculin**	**Paxillin**	**α-Actinin**	**β-Actin**	**α-Actin**	**SM1 SM2 myosins**

**Pristine**	100 ± 9	100 ± 14	100 ± 28	100 ± 15	100 ± 8	100 ± 4	100 ± 16
**Plasma**	134 ± 12	154 ± 17	98 ± 2	129 ± 28	77 ± 10	91 ± 10	69 ± 18
**Gly**	109 ± 12	140 ± 18	136 ± 23	50 ± 8^$^	68 ± 3	119 ± 6^C,^ *	78 ± 15
**PEG**	202 ± 16**^#, $^**	123 ± 8	152 ± 30	52 ± 10^$^	107 ± 8*	123 ± 6^$, C,^ *	128 ± 14
**BSA**	127 ± 10	151 ± 13	143 ± 27	37 ± 9^$^	95 ± 7*	115 ± 7	73 ± 17
**C**	112 ± 4	133 ± 9	104 ± 15	89 ± 8	111 ± 17*	87 ± 3	87 ± 16
**BSA + C**	201 ± 14^#,^ *	185 ± 14^#,^ *	114 ± 18	72 ± 12	110 ± 12*	111 ± 7	108 ± 13
**PS dish**	155 ± 29	113 ± 12	175 ± 37	93 ± 7	55 ± 9	89 ± 10	103 ± 16

Measured by ELISA per mg of protein. Means ± S.E.M. from three to seven experiments, each performed in duplicate or in triplicate. Absorbance values were normalized to the values obtained in cell samples from pristine PE, *i.e.*, given as a percentage of the values on pristine PE. ANOVA, Student-Newman-Keuls method. Statistical significance:

#, $, C, * : p ≤ 0.05 compared to the value on pristine PE, PE irradiated with plasma, PE irradiated with plasma and exposed to colloidal C particles, and polystyrene culture dish, respectively.

**Table 3.** Primary antibodies used for immunofluorescence staining (Immf.) and Enzyme-Linked Immunosorbent Assay (ELISA) of markers of adhesion and phenotypic maturation of VSMCs.

**Antibody against**	**Developed in, type**	**Company, Cat. No.**	**Dilution**	**Incubation**
**Chicken talin**	Mouse, monoclonal	Sigma a T 3287	Immf.: 1:200 ELISA: 1:500	Immf.: Overnight, 4 °C ELISA: 60 min, RT c
**Human vinculin**	Mouse, monoclonal	Sigma a V9131	Immf.: 1:200 ELISA: 1:400	Immf.: Overnight, 4 °C ELISA: 60 min, RT c
**Recombinant human paxilin**	Rabbit polyclonal	Chemicon b P1093	Immf.: 1:200 ELISA: 1:400	Immf.: Overnight, 4 °C ELISA: 60 min, RT c
**Chicken α-actinin**	Rabbit, polyclonal	Sigma a A2543	Immf.: 1:200 ELISA: 1:500	Immf.: Overnight, 4 °C ELISA: 60 min, RT c
**Synthetic peptide of α-smooth muscle actin**	Mouse, monoclonal	Sigma a A2547	Immf.: 1:200 ELISA: 1:400	Immf.: Overnight, 4 °C ELISA: 60 min, RT c
**Synthetic peptide of β-actin**	Mouse, monoclonal	Sigma a A 5441	Immf.: 1:200 ELISA: 1:400	Immf.: Overnight, 4 °C ELISA: 60 min, RT c
**Human smooth muscle myosin SM1 and SM2**	Mouse, monoclonal	Sigma a M7786	Immf.: 1:200 ELISA: 1:500	Immf.: Overnight, 4 °C ELISA: 60 min, RT c

a Sigma, St. Louis, MO, USA.; Czech Dealer: Sigma-Aldrich S.R.O., Prague, Czech Republic.

b Chemicon International Inc., Temecula, CA, USA.; Czech dealer: Scintilla S.R.O., Jihlava, Czech Republic.

c Room temperature (RT).

## References

[1] Bacakova L Svorcik V Rybka V Micek I Hnatowicz V Lisa V Kocourek F Adhesion and proliferation of cultured human vascular smooth muscle cells on polystyrene implanted with N^+^, F^+^ and Ar^+^ ions Biomaterials 1996 17 1121 1126 8718973 10.1016/0142-9612(96)85914-x

[2] Bacakova L Mares V Bottone MG Pellicciari C Lisa V Svorcik V Fluorine-ion-implanted polystyrene improves growth and viability of vascular smooth muscle cells in culture J. Biomed. Mater. Res 2000 49 369 379 10602070 10.1002/(sici)1097-4636(20000305)49:3<369::aid-jbm10>3.0.co;2-w

[3] Bacakova L Walachova K Svorcik V Hnatowicz V Adhesion and proliferation of rat vascular smooth muscle cells on polyethylene implanted with O^+^ and C^+^ ions J. Biomater. Sci.—Polym. Ed 2001 12 817 834 11587043 10.1163/156856201750411684

[4] Bacakova L Filova E Rypacek F Svorcik V Stary V Cell adhesion on artificial materials for tissue engineering Physiol. Res 2004 53 S35 S45 15119934

[5] Bacakova L Svorcik V Cell colonization control by physical and chemical modification of materials Cell Growth Processes: New Research Kimura D Nova Science Publishers, Inc. Hauppauge, NY, USA 2008 5 56

[6] Heitz J Svorcik V Bacakova L Rockova K Ratajova E Gumpenberger T Bauerle D Dvorankova B Kahr H Graz I Romanin C Cell adhesion on polytetrafluoroethylene modified by UV-irradiation in an ammonia atmosphere J. Biomed. Mater. Res. A 2003 67 130 137 14517870 10.1002/jbm.a.10043

[7] Svorcik V Rockova K Ratajova E Heitz J Huber N Bäuerle D Bacakova L Dvorankova B Hnatowicz V Cell proliferation on UV-excimer lamp modified and grafted polytetrafluoroethylene Nucl. Instr. Meth. B 2004 217 307 313

[8] Walachova K Svorcik V Bacakova L Hnatowicz V Smooth muscle cell interaction with modified polyethylene Biomaterials 2002 23 2989 2996 12069341 10.1016/s0142-9612(02)00029-7

[9] Turos A Jagielski J Piatkowska A Bielinski D Slusarski L Madi NK Ion beam modification of surface properties of polyethylene Vacuum 2003 70 201 206

[10] Wang Y Lu L Zhang Y Chen X Improvement in hydrophilicity of PHBV films by plasma treatment J. Biomed. Mater. Res. A 2006 76 589 595 16278866 10.1002/jbm.a.30575

[11] Kasalkova N Kolarova K Bacakova L Parizek M Svorcik V Cell adhesion and proliferation on modified PE Mater Sci Forum 2007 567–568 269 272

[12] Svorcik V Kasalkova N Slepicka P Zaruba K Bacakova L Parizek M Lisa V Ruml T Gbelcova H Rimpelova S Mackova A Cytocompatibility of Ar^+^ plasma-treated and Au nanoparticle-grafted PE Nucl. Instr. Meth. B 2009 267 1904 1910

[13] Mikulikova R Moritz S Gumpenberger T Olbrich M Romanin C Bacakova L Svorcik V Heitz J Cell microarrays on photochemically modified polytetrafluoroethylene Biomaterials 2005 26/27 5572 5580 15860214 10.1016/j.biomaterials.2005.02.010

[14] Rockova K Svorcik V Bacakova L Dvorankova B Heitz J Biocompatibility of ion beam-modified and RGD-grafted polyethylene Nucl. Instrum.Meth. B 2004 225 275 279

[15] Kotal V Svorcik V Slepička P Blahova O Sutta P Hnatowicz V Gold coating of PET modified by argon plasma Plasma Proc. Polym 2007 4 69 75

[16] Svorcik V Kotal V Siegel J Sajdl P Mackova A Hnatowicz V Ablation and water etching of poly(ethylene) modified by Ar plasma Polym. Degr. Stab 2007 92 1645 1651

[17] Chu PK Chen JY Wang LP Huang N Plasma-surface modification of biomaterials Mater. Sci. Eng 2002 R36 143 206

[18] Siegel J Reznickova A Chaloupka A Slepicka P Svorcik V Ablation and water etching of plasma treated polymers Rad. Eff. Def. Sol 2008 163 779 785

[19] Švorčík V Rybka V Stibor I Hnatowicz V Vacik J Stopka P Synthesis of grafted polyethylene by ion beam modification Polym. Deg. Stab 1997 58 143 147

[20] Švorčík V Hnatowicz V Stopka P Bačáková L Heitz J Ochsner R Ryssel H Aminoacids grafting of Ar ions modified PE Rad. Phys. Chem 2001 60 89 94

[21] Svorcik V Kolarova K Slepicka P Mackova A Novotna M Hnatowicz V Modification of surface properties of high and low density PE by Ar plasma discharge Polym. Degr. Stab 2006 91 1219 1225

[22] Orlandi A Ropraz P Gabbiani G Proliferative activity and alpha-smooth muscle actin expression in cultured rat aortic smooth muscle cells are differently modulated by transforming growth factor-beta 1 and heparin Exp. Cell. Res 1994 214 528 536 7925646 10.1006/excr.1994.1290

[23] Webster TJ Ergun C Doremus RH Siegel RW Bizios R Specific proteins mediate enhanced osteoblast adhesion on nanophase ceramics J. Biomed. Mater. Res 2000 51 475 483 10880091 10.1002/1097-4636(20000905)51:3<475::aid-jbm23>3.0.co;2-9

[24] Corning Cell Culture Surfaces: CorningR CellBINDR Polystyrene Surface Homepage. http://www.corning.com/lifesciences/us_canada/en/technical_resources/surfaces/culture/corning_cellbind_polystyrene.aspx (accessed July 6, 2009).

[25] Bacakova L Filova E Kubies D Machova L Proks V Malinova V Lisa V Rypacek F Adhesion and growth of vascular smooth muscle cells in cultures on bioactive RGD peptide-carrying polylactides J. Mater. Sci. Mater. Med 2007 18 1317 1323 17387596 10.1007/s10856-006-0074-1

[26] Filova E Bacakova L Lisa V Kubies D Machova L Lapcikova M Rypacek F Adhesion and proliferation of vascular smooth muscle cells on polylactide-polyethylene oxide copolymers with different content and length of polyethylene oxide chains Eng. Biomater 2004 7 19 21

[27] Horbett TA Principles underlying the role of adsorbed plasma proteins in blood interactions with foreign materials Cardiovasc. Pathol 1993 2 137S 148S

[28] Koenig AL Gambillara V Grainger DW Correlating fibronectin adsorption with endothelial cell adhesion and signaling on polymer substrates J. Biomed. Mater. Res. A 2003 64 20 37 12483693 10.1002/jbm.a.10316

[29] Koblinski JE Wu M Demeler B Jacob K Kleinman HK Matrix cell adhesion activation by non-adhesion proteins J. Cell Sci 2005 118 2965 2974 15976454 10.1242/jcs.02411

[30] Pignataro B Conte E Scandurra A Marletta G Improved cell adhesion to ion beam-irradiated polymer surfaces Biomaterials 1997 18 1461 1470 9426175 10.1016/s0142-9612(97)00090-2

[31] Kubova O Svorcik V Heitz J Moritz S Romanin C Matejka P Mackova A Characterization and cytocompatibility of carbon layers prepared by photo-induced chemical vapor deposition Thin Solid Films 2007 515 6765 6772

[32] Bacakova L Grausova L Vacik J Fraczek A Blazewicz S Kromka A Vanecek M Svorcik V Improved adhesion and growth of human osteoblast-like MG 63 cells on biomaterials modified with carbon nanoparticles Diamond Relat. Mater 2007 16 2133 2140

[33] Lesny P Pradny M Jendelova P Michalek J Vacik J Sykova E Macroporous hydrogels based on 2-hydroxyethyl methacrylate. Part 4: Growth of rat bone marrow stromal cells in three-dimensional hydrogels with positive and negative surface charges and in polyelectrolyte complexes J. Mater. Sci. Mater. Med 2006 17 829 833 16932865 10.1007/s10856-006-9842-1

[34] Han M Wen JK Zheng B Cheng Y Zhang C Serum deprivation results in redifferentiation of human umbilical vascular smooth muscle cells Am. J. Physiol. Cell. Physiol 2006 J291 C50 C58 16467401 10.1152/ajpcell.00524.2005

[35] Ezzell RM Goldmann WH Wang N Parashurama N Ingber DE Vinculin promotes cell spreading by mechanically coupling integrins to the cytoskeleton Exp. Cell Res 1997 231 14 26 9056408 10.1006/excr.1996.3451

[36] Sawada Y Sheetz MP Force transduction by Triton cytoskeletons J. Cell Biol 2002 156 609 615 11839769 10.1083/jcb.200110068 PMC2174068

[37] Van Amerongen A Wichers JH Berendsen LBJM Timmermans AJM Keizer GD Van Doorn AWJ Bantjes A van Gelder WMJ Colloidal carbon particles as a new label for rapid immunochemical test methods—Quantitative computer image-analysis of results J. Biotechnol 1993 30 185 195 7690572 10.1016/0168-1656(93)90112-z

[38] Lowry OH Rosebrough NJ Farr AL Randall RJ Protein measurement with the Folin phenol reagent J. Biol. Chem 1951 193 265 275 14907713

[39] Filova E Brynda E Riedel T Bacakova L Chlupac J Lisa V Houska M Dyr JE Vascular endothelial cells on two- and three-dimensional fibrin assemblies for biomaterial coatings J. Biomed. Mater. Res. A 2009 90 55 69 18481789 10.1002/jbm.a.32065

